# F-Waves – Physiology and Clinical Uses

**DOI:** 10.1100/tsw.2007.49

**Published:** 2007-02-02

**Authors:** Morris A. Fisher

**Affiliations:** Hines VAH and Loyola University Chicago Stritch School of Medicine, Chicago, IL, USA

**Keywords:** F-waves, late response, neuropathies, radiculopathies, peripheral nervous system, central nervous system

## Abstract

F-waves are low amplitude responses produced by antidromic activation of motoneurons. They may not appear after each stimulus and are inherently variable in latency, amplitude, and configuration. Meaningful analysis of F-waves requires an appreciation of these characteristics of F-waves as well as an understanding of their physiology. These features of F-waves as well as their physiology are reviewed. This is important since F-waves are one of the most frequently used studies in clinical neurophysiology and much of the controversies surrounding the use of F-waves relates to a failure to adequately consider the requirements of F-wave analysis. These requirements include the number of F-waves that need to be recorded, the parameters that should be evaluated, and the muscle from which the F-waves are recorded. If analyzed correctly, current reports would indicate that F-waves are the most sensitive and reliable nerve conduction study for evaluating polyneuropathies, can be abnormal in focal proximal nerve dysfunction, can be at least as sensitive as needle electromyography for defining lumbosacral radiculopathies, and can provide a meaningful physiological window into disorders of the central nervous system. Reports supporting these statements and their clinical relevance are discussed.

## INTRODUCTION

The F-wave is an interesting electrophysiological artifact produced by antidromic activation of motoneurons following distal electrical stimulation of motor nerve fibers. Because F-waves traverse the entire length of a peripheral nerve between the spinal cord and muscle, F-waves provide a means of examining transmission between stimulation sites in the arm and the leg and the related motoneurons (MNs) in the cervical and lumbosacral cord.

The F-wave is so named because it was originally studied in the small muscles of the foot[1] It is one of several responses that may follow the direct motor (M) response evoked by electrical stimulation of mixed or motor nerves. The most commonly observed and diagnostically useful of these responses, however, is the F-wave.

F-waves are commonly recorded using surface electrodes from small foot muscles (the abductor hallucis and the extensor digitorum brevis) and hand muscles (abductor pollicis brevis and abductor digiti minimi). Methods of recording are similar to that for standard motor conduction studies for the tibial and peroneal nerves in the legs or the median and ulnar nerves in the hands with stimulation at the ankles and wrists respectively. F-waves can also readily be recorded from calf muscles by stimulating the tibial nerve in the popliteal fossa. The recording can be made using a standard muscle-belly arrangement. When stimulating, the cathode is placed proximally to avoid anodal block of the antidromically traveling impulses. Although F-waves may be recorded following submaximal stimulation, they are routinely elicited following supramaximal stimulation for clinical purposes. Adequate analysis of F-waves requires recording a series of F- waves. Parameters commonly evaluated include latencies, differences between minimal and maximal latencies, durations, the percentage of discernible F-waves (persistence), amplitudes, and the number of F-wave that "repeat", i.e. the number of responses in a series of F-waves that are similar in latency, amplitude, and configuration.

An adequate recording and interpretation of F-waves requires an understanding of their physiology.

## Physiology of the F-Wave

F-waves are produced by antidromic activation ("backfiring") of MNs. F-waves are present in deafferented nerves in humans as well as other mammals[2,3,4]. Motor units (MUs) in F-waves are found only where the same MUs are found in the associated direct motor (M) response[5,6,7]. For any given MU, the shape and size of the associated motor unit action potential (MUAP) in the F-wave and direct response are identical. The antidromic activation of F-waves is further supported by single-fiber EMG studies showing that F-waves depend on activation of motor axons[8].

The probability that any one MU in a particular motor neuron pool will generate an F-wave is small[9]. Some stimuli in a train may not be followed by any F-wave. Where F-waves do follow the direct response, their shape and size usually changes from stimulus to stimulus (Figure 1) because the MUs and therefore the associated motor unit action potentials (MUAPs) which generate the F-wave, change with each successive stimulus[10,11,12]. The latency of the F-wave reflects the conduction time between the site of the stimulation of the peripheral nerve and the spinal cord (or brainstem in the case of the cranial nerves), for reactivation of the motoneurons (MNs).

## Physiological Factors Influencing the F-wave

Electrical stimulation of peripheral nerves is associated with antidromic activity in motor nerve fibers and orthodromic activity in sensory fibers. Both might influence the excitability of MNs and thereby the chance of an F-wave. For example, antidromic activity by invading collateral branches of the motor nerve fibers in the ventral horn will activate the inhibitory interneuronal Renshaw cells. Renshaw cells will transynaptically change the excitability of neighboring MNs. Renshaw cells are distributed widely throughout a MN pool[13], respond to increasingly intense stimulation by increasing their discharge frequency[14], and preferentially inhibit smaller MNs[15.16]. Other possible physiological influences of antidromic volleys include induction of a large enough field potential within the ventral horn to change the excitability of the MNs as well as inhibition by direct recurrent collaterals from MN to MN[17,18].

To have any influence on an F-wave discharge in a particular motor neuron, an antidromic volley in motor nerve fibers or orthodromic activity in sensory fibers would have to reach that motor neuron prior to its own antidromic activation. It is highly unlikely, therefore, that antidromic activity in more slowly conducting motor nerve fibers could influence the chance of an F-wave in more rapidly conducting MNs.

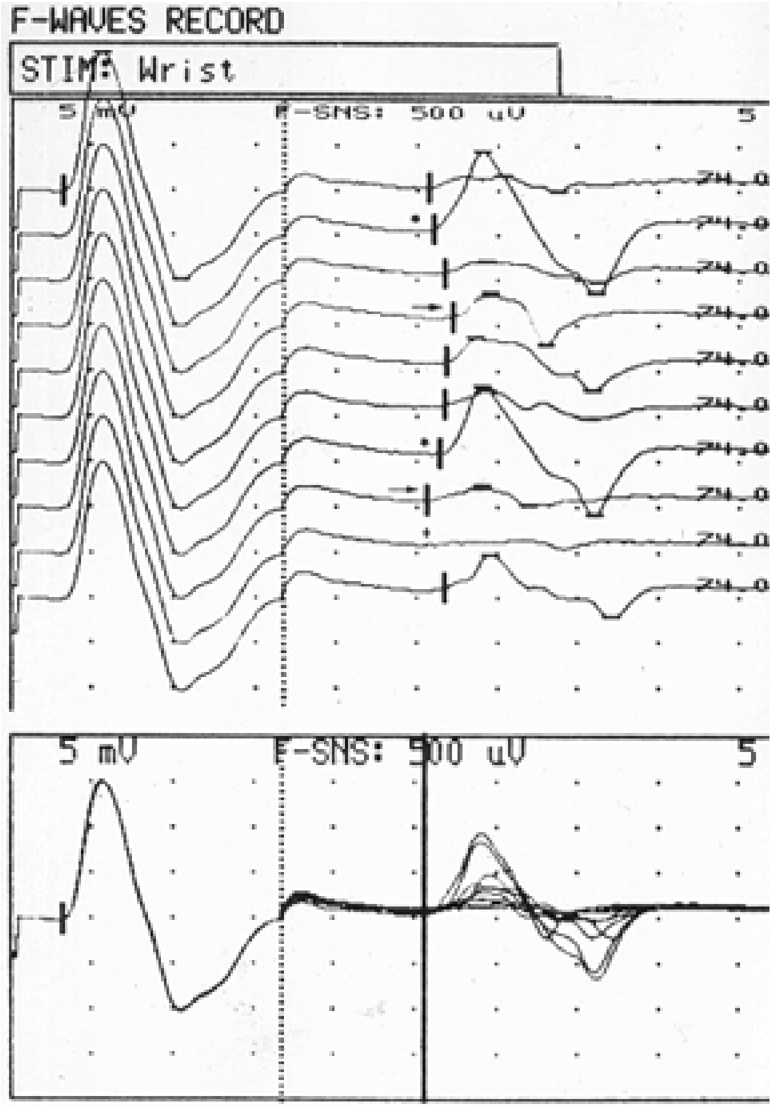

**Figure1.** F-waves (right) with the associated M-waves (left). The variability of F-waves is emphasized with the responses superimposed as shown below. Chronodispersion is the difference between the shortest and longest latencies. The persistence is 90%, i.e. one of the ten responses is absent. The two largest F-waves are repeater waves. Calibration per division: 5 mV for M waves, 500µV for F-waves; 5 ms. (Reproduced with permission, Fisher et al., 1994)

On-the-other-hand, antidromic activity in more rapidly conducting MNs might well influence the chance of an F-wave in more slowly conducting MNs. Even here, allowance would still have to be made for the time taken for the action potential to spread into the axonal collaterals within the ventral horn, synaptic activation of an interneuron, and finally synaptic activation of the MN. In theory, this should be possible given rise times for spinal cord MN excitatory postsynaptic potentials of at least 3 msec[19] and Renshaw cell influences on MN excitability following MN discharge in as little as 0.9 msec[13].

The precise manner by which an antidromic action potential triggers a second orthodromic action potential in the same MN has been examined only indirectly[20,21]. Nevertheless, it is reasonable to think that once it reaches the somadendritic (SD) membrane, the transmembrane currents associated with the antidromic action potential might be sufficiently strong to electrotonically reactivate the initial segment (IS). This requires that sufficient time has elapsed for the IS to recover from a refractory state from the preceding antidromic action potential. Once regenerated in the IS, the action potential then returns to the periphery to activate the corresponding MU thereby generating an F-wave.

This hypothesis for generation of an F-wave suggests that any factors tending to speed up the recovery of the IS or which delay and/or increase the magnitude of any antidromically induced depolarization of the SD membrane, might well increase the chance of an F-wave. By contrast, any factors which do the reverse might reduce the chance of an F-wave. F-waves may therefore be influenced by changes in “central excitability”. For example, MNs may become partially depolarized in some central motor disorders and the latter depolarization might be associated with reduced frequency (persistence) and amplitudes of the F-waves. The depolarization of the MNs may hasten the spread of the antidromic spike over the SD membrane and therefore reach the IS when it is still refractory such that an action potential is not generated. This is consistent with observations in both animals and man. MNs that discharge rarely with antidromic activation increase their firing with muscle activation; on the other hand, those MNs that fire frequently in the relaxed state will decrease their firing rate[9,22].

## Selectivity of Motor Units in the F-wave

Since there is a wide range in the physiological properties of MUs, the question of selection bias in the generation of F-wave is important. This is especially so since F-waves are composed of recurrent discharges of a very small proportion of the MN pool. Selection could affect the conduction velocities and therefore latencies of the MUs in the F-wave and whether the range and distribution of conduction velocities in the F-wave are representative of the motor nerve as a whole. At the moment, there are no certain answers to such questions but discussion of these issues is important to enhance an understanding of F-waves.

The diameters of the largest nerve fibers in the ventral roots are roughly twice those of the smallest ventral root fibers[23]. This would be consistent with the fastest conduction velocities in alpha motor axons being twice that of the slowest. This has been found in at least some studies reporting pooled data from human motor fibers (see Table 4,[24]). The relevance of this argument has been questioned because the range of F-wave conduction velocities in individual subjects is less than the range based on pooled data (Doherty et al, 1994), but ranges of conductions at least twice the 15-20% normally observed in F-waves have been noted in human motor fibers in individual subjects[25]. These observations suggest that there may be some bias toward the faster conducting MNs in the F-wave.

If enough F-waves are analyzed, latencies using supramaximal stimuli are normally distributed[26]. At the same time, the number of F-waves analyzed is usually small – normally less than 10. This would suggest that minimum F-wave latencies might be so variable as to be of little clinical value. This is not true and again suggests a selective activation of the largest, fastest conducting motor units in F-waves.

Several studies have suggested that there is no bias in the selection of MUs in the F-wave[27,28]. Furthermore, using twitch force as a measure of motor unit size, MUs of all sizes were reported to produce F-waves[29]. At the same time, larger MUs with the stronger twitch tensions were said to be “more subject” to F-wave production. The absence of any bias was also the conclusion from studies using a method for determining the conduction velocities of single motor nerve fibers using threshold stimulation at different points along the course of a nerve[24,28]. Studies of the F-wave using collision support the full range of MNs are represented in the F-wave[30]. This conclusion, however, has been questioned[31] and selective activation of larger MNs has been reported using a similar technique[32]. All studies supporting absence of bias in the selection of MUs in F-waves have been performed at submaximal stimulation. The relevance of such studies to others where supramaximal stimuli were used and activation of Renshaw cells might influence the chance of F-waves at this point is unclear.

Despite concerns about the adequacy of the small numbers of F-waves used to determine the shortest latency F-wave, changes in the latter latency are very similar to changes in maximal evoked response latencies when the sites of stimulation are changed[33,34,35,36]. The latter finding lends support for the idea that there may be some form of selective activation of the largest, fastest conducting motor units in F-waves and is consistent with findings based on studies of single MUs in F-waves as well as analysis of F-wave conduction velocities[36]. In the opinion of the author the weight of the evidence favors some bias toward preferential activation of larger MUs in F-waves. Fortunately, the observed variability in F-wave latencies is not so random or great as to preclude their clinical use.

## METHODOLOGY

F-waves are ubiquitous in distribution. F-waves recorded from distal muscles in the limbs are usually clearly separated from the direct M-potential. With more proximal stimulation, however, F-waves may be obscured by the preceding direct M-response due to the shorter latency of the F-waves. Collision techniques may be employed to cancel the effect of the direct M potential on the F-waves and make it possible to measure the latencies of the F-waves with proximal recordings[37].

F-waves are usually recorded using surface electrodes in a belly-tendon fashion with the active electrode positioned over the innervation zone of the target muscle. When recording from calf muscles, both electrodes should be positioned over the muscle belly to reduce any pickup of late responses from more distant muscles. As when recording H reflexes from the calf muscles, the recording electrode can be positioned on the soleus one-half the distance between the stimulation site in the popliteal fossa and the superior aspect of the medial malleolus[38]. The stimulating cathode should be proximal to the anodal electrode to avoid anodal block. F-waves may be affected by a previous conditioning stimulus and F-wave. Recovery curves for F-waves have been reported[39]. For this reason the rate of stimulation should be less than 0.5 Hz.

F-waves may be present following submaximal sitmulation, but F-waves are more prominent with supramaximal stimulations (i.e., 25% above that required for the maximum M-wave) since the amplitudes of the F-waves as well as the frequency of occurrence (persistence) increase as the stimulus intensity increases. Supramaximal stimulation also provides a physiologically definable and uniform environment in which F-waves will occur. In the author's opinion, therefore, supramaximal stimulation should be used routinely for collecting F-waves. Given the relatively small sizes of most F-waves, the associated supramaximal M waves generally need to be recorded at a lower gain.

There has been interest in submaximal stimulation of F-waves, which produces less discomfort. Aside from the question as to how many stimuli may then be required to obtain meaningful data, subamaximal stimulation alters F-wave parameters except possibly for latency and the duration of the responses[40]. Weak contraction of the recording muscle can increase the chance and size of the F-wave but may alter F-wave parameters and contaminate the recordings with H reflex responses[41,42].

### Analysis of F-waves

F-wave latencies reflect the conduction times between the site of stimulation and the spinal cord, the time for reactivation of the MN, and the time for the centrifugally conducted action potential to activate the MU. Individual F-waves are generated by the recurrent discharges from one to at the most a few MUs whose associated MUAPs and F-wave latencies differ from one another. Variation in which individual MUs generate F-waves is responsible for the changes in the size, shape and latency of the composite F-wave. These factors account for the small size of the F-wave relative to the size of the direct M-potential (generally < 5%)and the inherently variable size, latency, and shape of the F-wave from stimulus to stimulus (Figure).

There is uncertainty about the "turnaround" time for individual MUs. This time is said to be of the order of 1 ms based on a statement by Eccles[20]. However this time has never been directly measured. In invertebrates the turnaround time is appreciably longer[43] but the relevance of the latter values to mammals is unclear. In humans, variations in latency of identical F-waves using single fiber recordings have been small (<70μs)[9]. Using the more common techniques for obtaining F-waves including supramaximal stimulation, latency variations of 3 ms have been reported[44].

The inherent variability of F-waves dictates that a sufficient number of F-waves are recorded to insure that accurate, reproducible data be recorded. The most common mode of assessing F-waves has been to collect a sample of 10 or more F-waves and then to measure the shortest latency F-wave. However it may be difficult to identify the latencies of some F-waves because the F-wave is superimposed on the steep negativity following the preceding direct M-potential or the initial slope of the F-wave is too gradual to allow the operator to clearly identify the onset of the F-wave. In addition individual F-waves may overlap and be confused with axon reflexes and A-waves[45,46]. As is the case for F-waves, axon reflex latencies also diminish as the stimulus is moved proximally. Unlike F-waves however, axon reflexes may be blocked as the stimulus intensity is increased due to "collision" in the efferent limb of the reflex. A-waves will persist with increasing stimulus intensity and may be superimposed on F-waves or occur before or after the F-waves.

A more reliable method of comparing latencies in F-waves is to determine the mean latency. The latter does not depend on identifying and measuring the latency of a single F-wave, better reflects the range of F latencies, and is more reproducible than minimal latencies. For these reasons, mean latencies have been recommended by multiple studies[47,48,49,50,51,52,53]. Chronodispersion refers to the difference between the minimal and maximal latencies[54] and thereby reflects the range of latencies in a series of F-waves. F-waves are readily discernible if greater than 40μV in peak-to-peak amplitude, and persistence refers to the percentage of such responses following a series of stimuli. Because of the variability of F-waves, the amplitudes of F-wave are best measured as mean values and related to the amplitude of the maximum M-potential, i.e. the mean F/M ratio. Total durations, i.e. from onset to return to baseline, have been measured. Identical responses in a series of F-waves are called repeater waves.

F latencies have been converted to conduction velocities. This translates F latencies into values that can be directly compared with equivalent more distal motor conduction velocities. The range of such conduction velocities (F tacheodispersion) determined from F-waves may be helpful for assessing conduction in peripheral neuropathies[55]. F-tacheodispersion, however, has the disadvantage of introducing potential additional measurement errors, namely measurements of distance and correct allowances for the turnaround times of MUs.

When recording from hand, calf, and foot muscles, upper limits of normal for minimal latencies of 31, 37, and 60 ms respectively may be used based on either 95$^{th}$ percentile or mean $\pm$ 2 SD values[47,56,57,58,59]. The values for mean latencies are about 2 ms greater. F-wave latencies, however, will vary with limb length and, to a lesser degree, age. Further refinement for predicting F-wave latencies is possible by adjusting the foregoing values for height[56,57,58,59] or using formulas that include height or limb length and age. Regression equations for minimal and mean F-wave latencies using these variables are available[47,60,61,62]. Predicted F latencies using these preceding adjustments are easily and quickly determined using hand calculators.

Based on analysis of normative data, we have found the upper limit of normal for side-to-side differences using mean latencies is 2 ms in hand muscles, 3 ms for the calf, and 4 ms for foot muscles. These values are comparable with other published reports[56,57,58,59]. Normative values for F-wave durations have been reported[49,53,63,64,65]. Persistence will vary depending on the muscle studied and is higher in antigravity muscles, namely the extensors in the legs and the flexors in the arms, than their antigravity antagonists[66,67]. Although the range of normal values is high, persistence in antigravity muscles such as the abductor pollicis brevis, calf and abductor hallucis muscles usually exceeds 80% while for antigravity antagonists such as the extensor digitorum communis, tibialis anterior, and extensor digitorum brevis the values range between 30-40%. Published upper limits of normal for chronodispersion have varied[53,54,55,60,62,63,68,69] and may depend on the number of F-waves recorded[26].

**Table 1**

***Latencies***

| | Minimal | Mean |
|---|---|---|
| APB (median) | (-3.5)+0.063a+0.37d | 0.04+0.056a+0.35d ±2msec |
| ADM (ulnar) | (-1.5)+0.056a+0.36d | (-0.40)+0.063a+0.35d ±2msec |
| Soleus (tibial) | 15.6+0.051a+0.31d | 10.5+0.092a+0.43d ±3msec |
| EDB (peroneal) | 46.3±3.2(ht 147-160) | (-26.4)+0.112a+0.39ht* ±5msec |
| | 49.3±3.8(ht 163-175) | |
| | 52.8±4.2(ht 178=193) | |
| AH (tibial) | 47.3±3.6(ht 147-160) | (-28.5)+0.131a+0.414ht* ±5msec |
| | 50.6±3.7(ht 163-175) | |
| | 55.4±4.2(ht 178-193) | |

Upper limit of normal for mean F-wave latency differences between sides are 2 msec for the hand, 3 msec for the soleus, and 4 msec for the foot.

*Mean latency – M latency
•APB-abductor pollicis brevis; ADM-abductor digiti minimi; EDB-extensor digitorum brevis; AH-abductor hallucis
•a-age in years; d- distance in cm (for APB and ADM, C7 to superior aspect radial styloid; for soleus, mid popliteal fossa to superior aspect of the medial malleolus); ht- height in cm
•latencies and regression equations shown with standard deviations
•regression equations for APB, ADM, and soleus from [26]; for EDB and AH from [62]

***Chronodispersion****
APB 6.2 msec
ADM 5.5 msec
Soleus 7 msec
EDB 9.5 msec
AH 9.3 msec

*upper limit of normal

***Amplitudes (mean F/maximum M [mF/M] ratios)****
APB 22±10**
Soleus 25±12

*mean F amplitude in µV, maximum M in mV
**mean±SD

Based on the published reports, false positive values are unlikely if the upper limits of normal are 6.2 ms for the abductor pollicis brevis and adductor digiti minimi, 7 ms for calf muscles, and 9.5 ms for the extensor digitorum brevis and abductor hallucis. Responses similar in amplitude, configuration, and latency may be identified in a series of F-waves, namely repeater waves. The maximum frequency of a particular F-wave in small hand muscles is normally 10% or less[70,71], but the percentage of such repeater waves may be higher[72]. Using mean F amplitude and maximum M values, normal F/M amplitude ratios in our laboratory have been 2.2 $\pm$ 1.0% recording from the abductor pollicis brevis and 2.5 $\pm$ 1.2% recording form the soleus, equivalent to an upper limit of normal of about 5%. Normal values for F-wave parameters used in our laboratory are shown in Table 1.

Because of the inherent variability of F-waves sufficient F-waves must be collected to provide representative data. Ten stimuli yielding anywhere from 7-10 F-waves probably suffice for most studies of persistence and latencies. However, 20 or more stimuli providing anywhere from 16-20 F-waves may be needed for accurate measurements[26,50,51,52,73,74]. The issue of an adequate number of stimuli as well as the mode of analyzing F-waves is important when comparing relatively small latency differences between sides such may be necessary in radiculopathies. A series of 20 F-waves is also adequate for the measuring mean F/M amplitude ratios and the percentage of repeater waves. As few as two F-waves may be adequate for establishing an abnormal chronodispersion if the separation in latency between these two responses is greater than normal. Accurate measurement of chronodispersion requires more than 20 stimuli, possibly as many as 50–60[26,55]. To determine the number of individual repeater waves requires at least 100 stimuli.

## CLINICAL APPLICATIONS OF THE F-WAVE

### Peripheral Neuropathies

Evaluation of peripheral neuropathies is one of the most frequent indications for electrodiagnostic examinations. Polyneuropathies imply involvement of multiple nerves. Traditionally, these have been divided into those in which the injury is considered primarily axonal in contrast to those where the primary insult is to the myelin. Axonal neuropathies are characterized by decrease in evoked response amplitudes, relatively limited conduction slowing, and abnormalities on needle examination of muscles due to disruption of the normal connection between the nerve and muscle. In demyelinating neuropathies, there is more prominent slowing of conduction. At times in demyelinating neuropathies, there is temporal dispersion of the responses as well as conduction block, namely absence of conduction in a nerve due to focal disruption of myelin.

The latencies of F-waves are characteristically prolonged in neuropathies and may be abnormal even when peripheral motor conduction studies are normal[63,75,76,77,78,79]. F-waves may also be more sensitive than conventional motor conduction studies in axonal neuropathies[61]. F-wave latencies have been reported to be the most stable and reliable measurement for sequential nerve conduction studies in the same subjects[80] and are the most sensitive nerve conduction parameter in patients with diabetic neuropathies[81]. This may be true because F-waves are affected by dysfunction along the entire course of a nerve. Prolonged F-wave latencies exceeding 150% of the upper limit of normal have been considered as very suggestive of demyelinating neuropathies, as has the absence of F-waves in the presence of relatively preserved maximum M-potentials[61].

F-wave parameters other than latency can provide additional valuable electrophysiological information. For example the percentage of repeater waves is increased in neurogenic disorders, especially those associated with atrophy[44]. Increases in repeater waves have been reported to be a sensitive indicator of carpal tunnel syndrome[72]. This study was based on series of 100 F-waves - a correct but practically limiting approach. Increases in the durations of F-waves may also be an early sign in diabetic neuropathie [65]. Mean F/M amplitude ratios may be increased in neuropathies. This is most characteristic of axonal injuries [44] and is consistent with the increased amplitudes of the F-waves and reduced amplitudes of the maximum M-potentials, so often a feature of these neuropathies.

Other F-wave abnormalities may also be helpful for evaluating polyneuropathies. This is particularly true for acute and chronic acquired demyelinating neuropathies where F-wave abnormalities have been reported in greater than 90% of nerves studied[61,63]. Abnormal F-wave latencies have been found in no more than 50% of these nerves. Other F-wave findings have included absent responses in about 50% of the nerves and abnormal chronodispersion or persistence present in about 25% to 50%. These F-wave findings were frequently the only F-wave abnormality in a particular nerve[63]. F-wave abnormalities have been at least as frequent as motor conduction study abnormalities[61]. The high sensitivity of F-waves in acquired demyelinating neuropathies such as the Guillain-Barré syndrome[34,75,82] is consistent with both the demyelination and the focal proximal injury in these patients.

Characteristic F-wave findings in axonal and demyelinating neuropathies are summarized in Table 2.

**Table 2**
**Polyneuropathies – Characteristic Findings**

*Axonal*

- Mildly prolonged F-wave latencies
- Increased mF/M values
- Increased repeater waves

*Demyelinating*

- Prominently prolonged F-wave latencies (i.e., >150% of the expected value)
- Decreased persistence
- Absent F-waves with relatively preserved M-waves

## F-waves in Proximal Nerve Lesions

As noted above, F-wave latencies are possibly the most sensitive and reliable nerve conduction measurements in patients with neuropathies. F-latency prolongation has also long been described in patients with focal proximal nerve lesion[83,84]. As also noted above, F-wave abnormalities have a high sensitivity in acquired demyelinating neuropathies in which focal proximal demyelination may be the main pathological feature.

## F-waves in Radiculopathies

Experimentally, root injury is due to compression and inflammation. This would produce demyelination, and slowing of nerve conduction can be demonstrated[85]. As such, one might expect that F-waves might also be of value in the electrophysiological evaluation of radiculopathies. In fact, the use of F-waves in the radiculopathies has been controversial.

### *Criticism*

The use of F-waves in radiculopathies has been criticized because the injury may not involve all of the motor axons in that particular root. This argument would be reasonable if the only F-wave parameter that could be analyzed were the minimal latency. F-waves in fact are uniquely qualified to analyze radicular injury where there may be axons that are injured while others may remain intact. F-wave parameters that can measure this range of normal versus abnormal conductions include mean, median, and maximum latencies as well as chronodispersion. The same argument applies to those who argue that F-waves cannot be used because the recording muscles may have multiple root innervation. These types of critical arguments are probably less relevant today than it might have been in the past. Because of the current diagnostic quality of radiographic studies, most patients now having electrodiagnostic (EDX) examinations for lumboscral may not have focal root injury but rather spinal stenosis with associated multiple root dysfunction.

Another theoretical criticism of the use of F-waves in lumbosacral radiculopathy has been based on the concept of "dilution"; namely, the relatively small latency delay associated with nerve root compressed would be obscured by the much longer F-wave latency. This argument ignores the reliability and reproducibility of F-wave latencies discussed above and therefore, if analyzed appropriately, the ability to compare differences between sides. The argument also ignores the additional information that may be obtained by analyzing F-wave parameters other than latency. Modeling F-wave latency changes in radiculopathies using signal detection theory indicates that absolute F-wave latency does not influence the accuracy of detecting focal lesions[86]. This makes the theoretical argument for the "dilution" hypothesis irrelevant. The important variable in fact is variance. This emphasizes the importance of using techniques that decrease the variance of F-wave latency measurements such as use of mean rather than minimal latency values.

Finally, the use of F-waves in radiculopathies has been criticized because the data overlaps with that obtained with needle electromyography (nEMG). This is not necessarily supported by available reports, and it is not what one might expect based on the pathophysiology of root injury. nEMG requires axonal injury while F-wave abnormalities could occur with demyelination. At a more fundamental level, the argument misses the point. The important question is whether F-waves could be helpful in patients with radiculopathies. If this is true, then as noted in an early discussion of this question, F-wave studies are indicated where the information could be meaningful for the diagnosis of a radiculopathy[87]. This would be true, for example, where F-waves provide the only physiological evidence of injury to anterior primary rami in such patients.

The most commonly cited article criticizing the use of F-waves in radiculopathies is that by Aminoff and colleagues[92] and may be the only study cited in reviews critical of F-waves in radiculopathies[88]. This study evaluated 28 patients with "clinically unequivocal lumobsacral radiculopathy" (L5 and/or S1), 4 of whom did not have confirmatory radiographic studies. The authors state that the diagnostic yield of F-waves was disappointing, namely in only 5/28 patients and all of these had nEMG abnormalities. These conclusions were based on F-waves recorded only from the extrensor digitorum brevis muscle (EDB) following only 10 stimuli and based on normative values that did not include corrections for height or limb length nor age. Any abnormality was therefore based on absolute latency values using minimal latencies predictably from only 3-4 F-waves in the antigravity antagonist EDB. This methodology of F-waves analysis by current standards would be considered inadequate and the conclusions meaningless. Furthermore, the predominant innervation of the EDB is L5 and yet more than 75% of the patients had S1 lesions.

## *Defense*

Up to 90%of radiculopathies occur at the lumboscacral level and up to 80% involve the L5 and/or S1 roots[88]. These roots innervate muscles commonly used for F-wave recordings, namely calf or small foot muscles. By contrast, close to 90% of cervical radiculopathies involve the C5, C6, or C7 root[89]. These roots do not supply the C8, T1 innervated muscles commonly used for F-wave recordings and are therefore not readily subject to F-wave. As such, meaningful information about the use of F-waves in cervical radiculopathies is not available.

Based on prolonged latencies or abnormal side-to-side differences, sensitivities of about 50-80% were reported about 30 years ago for F-waves in the evaluation of lumbosacral radiculopathies[90,91]. Sensitivities were particularly high for S1 radiculopathies when recording from calf muscles, and there were patients with normal nEMG studies and abnormal F-waves. These studies were based on analysis of minimal F-wave latencies following a limited number of stimuli (i.e., 10), and the sensitivity of F-waves in radiculopathies was subsequently questioned[88].

More recent studies using F-wave parameters in addition to minimal F-wave latencies have reported a sensitivity in L5/S1 radiculopathies comparable to that for nEMG. In 96 patients with L5/S1 radiculopathies, over 40% of these patients had clinically relevant absent or prolonged latency F-waves and 76% had abnormal chronodispersion[93]. In a similar series of patients with L5/S1 injury and using similar F-wave parameters, needle EMG studies were abnormal in 70% while F-wave abnormalities were found in 69% [94]. F-wave abnormalities were found in 13 of the 23 patients where the only nEMG denervation was in the paraspinal muscles thereby providing unique evidence for injury to the anterior rami. In 95 patients with L5, S1, or L5 and S1 root lesions con firmed by surgery (78) or myelography, F-waves were abnormal in 70% of the patients, nEMG 77%[95]. The F-wave parameters evaluated included chronodispersion and mean F-wave duration. Using similar criteria for normal versus abnormal F-waves, improvement in F-wave parameters has been correlated at a statistically significant level with recovery in strength following surgery[96]. In 20 patients with surgically verified L4, L5, or S1 radiculopathies, nEMG was abnormal in 12 and F-wave abnormalities were noted in 8; in 3 of these 8 patients, nEMG was unrevealing. F-wave abnormalities in this study were based on prolonged F latencies, abnormal latency differences between sides, and/or abnormal persistence[97]. Based on a study of 42 patients with "acute" (less than 4 weeks) lumbosacral radiculopathies, F-wave abnormalities including chronodispersion and persistence were as sensitive as nEMG in patients with weakness and more in patients without weakness[98]. Wells and colleagues[99] used a multiparameter, computer analyzed composite measurement to evaluate lumbosacral root compression. This measurement included five F-wave latency parameters. The study was blinded, prospective, and had a control group. Using this composite approach, the authors reported a diagnostic specificity of 84.3% and a sensitivity of 83.3%.

Using increased minimal latencies and/or chronodispersion, 69% of the tibial or peroneal nerves studied in patients with spinal stenosis had abnormal F-waves while this was true in only 24% of the nerves in patients with L5/S1 root compression syndromes. In both sets of patients, however, 3 minutes of standing produced an abnormal increase in F-wave chronodispersion[100]. In some patients, this increase in chrondispersion with standing was as much as 8 ms. This study is consistent with F-waves having meaningful diagnostic utility in spinal stenosis and that focal radicular injury can produce discernible changes in F-waves. This has been supported by two recent reports[101,102].

In a methodologically reasonable study evaluating 24 patients with clinically and radiologically L5 root injury, reportedly surgically confirmed, the authors concluded that EDB F-waves did not provide meaningful additional information. Data examined included F-wave latencies and persistence. Analysis of their data, however, in fact indicates that there was a meaningful decrease in the mean persistencies on the affected side in comparison to the unaffected side ($p<0.02$).

## Conclusion

There are therefore no convincing theoretical arguments and no convincing studies indicating that F-waves cannot be helpful in lumbosacral radiculopathies. Theoretical considerations and the weight of clinical studies indicate that F-waves can be abnormal in L5/S1 radiculopathies and may have sensitivity comparable to nEMG. For this to be true, however, F-waves need to be analyzed appropriately. Minimum F-wave data alone is not adequate, and multiple F-wave parameters need to be evaluated. As with any EDX study (or studies)[103], F-waves cannot be used as the sole evidence for a radiculopathy. The current evidence, however, would support the usefulness of F-

waves in the EDX evaluation of radiculopathies where such evidence of injury to the anterior rami could be helpful.

## F-waves and Motor Unit Number Estimates

Using multiple point stimulation, single MUs in F-waves can be identified[104]. Based on analysis of series of F-waves following 300 stimuli at stimulus intensities producing M waves 10-50% of maximal, an automated, computerized method has been described for motor unit estimates using F-waves to determine the average size of MUAPs generated by single MUs[105]. F-waves, which were identical to one another in shape, size and latency, were considered to represent MUAPs generated by single MUs. The distribution of MUAP sizes and motor unit number estimates obtained with this method were similar to those obtained with multiple point stimulation and showed age-related losses of MUs.

## Central Nervous System Disorders and F-waves

F-waves provide a physiological window into a segmental motoneuron pool excitability, even if not necessarily for short-term changes[106]. In patients with upper motor neuron syndromes due to central nervous neuron (CNS) lesions, antidromically activated motor neurons fire more frequently than those in normal individuals[9]. At the same time, this is complicated by the fact that frequently backfired motor neuron will discharge less frequently with activation by muscle contraction, whereas motor neurons that discharge infrequently will increase their firing rate. These observations are consistent with F-wave studies in deafferented animals[4]. At times, therefore, increased central excitability results in decreased discharge of larger motor neurons in an F-wave owing to blockage at the still refractory initial segment of the motor neuron. Despite this complexity, analyses of F-waves are a valuable technique for monitoring central motor neuron excitability.

In patients with CNS lesions, the normal, relatively increased prominence of F-waves in resting extensor muscles compared with flexor muscles may be disrupted. F-wave amplitudes and persistence are decreased in clinically involved limbs, and this finding is compatible with decreased central excitability in patients studied early after unilateral cerebrovascular lesions, a period when decreased tone and reflexes are common findings[107,108]. Similarly, F-waves are absent in patients with spinal shock due to injury and are decreased in prominence in similar patients without spinal shock[109]. F-wave amplitudes and persistence can be decreased by cerebellar stimulation, which is consistent with increased cerebellar inhibitory outflow[110]. By contrast, F-wave persistence and average F-wave amplitudes, as well as F/M ratios, are increased in patients with "spasticity." Huge F-waves—as large as 75 percent of M-wave amplitudes—have been found in chronic tetanus[111]. These waves were associated with a shortened or absent silent period, compatible with the failure of inhibition of Renshaw cells and thereby indirectly supporting a role for Renshaw cell activity in F-wave discharge.

In patients with upper motor neuron syndromes, F-wave latencies may be prolonged and durations and amplitudes are increased[112]. These data are consistent with the discharge of a greater number of smaller, slower-conducting motor units owing to increased central excitability while larger motor neurons are blocked because of too rapid activation, as discussed earlier. Correlations between F-wave latencies, durations, and amplitudes are also disturbed in patients with motor disorders of central origin[112]. These data suggest that analyses of F-waves could be used to define different abnormal states of the motor system for clinical purposes.

Knowledgeable use of F-waves requires an understanding that these responses originate at the interface between the central and the peripheral nervous systems. F-wave studies can provide physiologic insight into that interface. F/M amplitude ratios, for example, are increased in patients with polyneuropathy as well as those with spastic hyperreflexia[113] . Log F/M values are normally directly correlated with neuromuscular efficiency as defined by twitch tension/M-wave amplitudes. This relationship is disturbed most prominently in patients with CNS abnormalities, but also in patients with peripheral nerve dysfunction[114].

## CONCLUSION

The F-wave is an important tool for evaluating the normal and abnormal physiology of the peripheral and central nervous systems. F-waves, however, need to be used thoughtfully with an understanding of their physiology. Such efforts can provide meaningful clinical information. F-waves have an established role in the electrodiagnostic evaluation of peripheral nerve dysfunction. In polyneuropathies, F–waves can both help establish that a neuropathy is present as well as help characterize its nature. Despite criticisms, the current evidence supports F-waves having a role in the electrophysiological evaluation of lumbosacral radiculopathies. F-wave analysis may be useful for motor unit estimates. F-waves provide a readily accessible "window" into the central nervous system with preliminary evidence that this could be a worthwhile approach for evaluating central nervous system disorders.
